# Uncovering the Uncommon: Institutional Insights Into the Clinical and Epidemiological Characteristics of Rarer Forms of Bladder Cancer Beyond Transitional Cell Carcinoma

**DOI:** 10.7759/cureus.40879

**Published:** 2023-06-23

**Authors:** Anshuman Singh, Anupam Choudhary, Vivek Pai, Kasi Viswanath, Surag K R, Goli V Abhishek, Arun Chawla, Padmaraj Hegde

**Affiliations:** 1 Urology, Kasturba Medical College of Manipal, Manipal Academy of Higher Education, Manipal, IND

**Keywords:** bladder carcinoma, tcc, transitional cell carinoma, non-urothelial bladder cancer, bladder cancer

## Abstract

Introduction

Non-transitional cell carcinomas of the bladder (NTCCB) represent a significant clinical challenge due to their rarity, heterogeneity, and poor prognosis. Despite their poor prognosis, the treatment of NTCCB has historically been based on the same principles used for transitional cell carcinomas (TCCs). Our study focuses on the management of non-transitional cell carcinomas and aims to identify areas where treatment outcomes can be improved based on our institutional experience.

Materials and methods

A retrospective analysis of patients with NTCCB who presented at Kasturba Hospital Manipal was conducted between 2012 to 2021. Patient data were collected, and demographic characteristics, presenting symptoms, history of other primary malignancies, comorbidities, location of the tumour, stage at presentation, histopathological subtype, site of systemic metastasis, and primary treatment given were analyzed descriptively. Median overall survival was determined by calculating the time from the initial diagnosis to the date of death.

Results

Among 31 patients with NTCCB, 15 (48%) presented with metastatic disease, five (16%) with locally advanced disease, and 11 (36%) with localized disease. The most common histopathological subtypes were squamous cell carcinoma and adenocarcinoma, as noted in 14 (45.2%) and 13 (41.9%) patients, respectively, followed by neuroendocrine tumours in two (6.5%), extra-adrenal phaeochromocytoma in one (3.3%), and sarcomatoid carcinoma in one (3.3%) patient, respectively. The lung was the most frequent site of systemic metastasis as noted in six (40%) patients, followed by the liver and skeletal system in three (20%) patients each, peritoneum in two (13.3%), cerebral cortex in one (6.7%), and non-regional lymph nodes in one (6.7%) patient. The primary treatment given included palliative chemotherapy in 14 (45.2%) patients, radical cystectomy with ileal conduit in 10 (32.3%), neoadjuvant chemotherapy only in four (12.9%), partial cystectomy in one (3.2%), pelvic exenteration with ileal conduit in one (3.2%), and peritoneal debulking with palliative chemotherapy in one (3.2%) patient. The overall median survival was 15 months, with a one-year survival rate of 67.4%.

Conclusion

NTCCB exhibits aggressive clinical behaviour and presents with nonspecific clinical features in the early stages, often leading to late diagnosis and an advanced tumour stage at presentation. Multi-institutional studies with larger patient cohorts are needed to recommend best clinical practices for early detection and optimal treatment strategies to improve patient survival.

## Introduction

Carcinoma of the bladder is one of the most common cancers of the urinary tract worldwide [1]. The majority (90%) of bladder carcinomas are transitional cell carcinomas (TCCs) [2]. Non-transitional cell carcinomas of the bladder (NTCCB) are rare and account for 10% of cases worldwide, known for their aggressive nature [3]. According to the current literature, the most common types of non-TCC bladder carcinoma are squamous cell carcinoma (SCC) (3-5%), followed by adenocarcinoma (0.5-2%) and small cell carcinoma (<0.5%) [1]. Additionally, sarcomatoid carcinoma, neuroendocrine tumor, clear cell carcinoma, and lymphoma are extremely rare variants, accounting for less than 0.1% of cases. These tumors are so rare that some urologists may never encounter these in their practice [4].

Based on the etiology of the cancer, SCC of the bladder can be classified as bilharzial and non-bilharzial. These variants differ in terms of epidemiology, pathogenesis, and clinicopathological characteristics. Bilharzial SCC is associated with Schistosomiasis haematobium and is more commonly observed in Egypt and other African countries [5,6]. On the other hand, non-bilharzial SCC is seen in patients with bladder stones, recurrent urinary tract infections, chronic indwelling catheters, and those with prolonged exposure to cyclophosphamide [7]. These conditions lead to chronic irritation of the bladder, resulting in urothelial metaplasia, and predisposing to SCC [5]. Adenocarcinoma of the urinary bladder can be of urachal and non-urachal origin [8]. Risk factors for adenocarcinoma include male gender, ectopia vesicae, cystocele, and villous adenoma [8]. While there is some variability in the definition of adenocarcinoma in the literature, the pathological hallmark of this tumor is the presence of neoplastic cells that form glandular structures resembling colonic adenocarcinoma (enteric type) and/or produce large amounts of intra- or extracellular mucin (mucinous type) [8,9]. Adenocarcinoma is associated with a worse prognosis due to the higher stage at presentation [10].

Sarcoma is the most common mesenchymal malignancy of the bladder, with leiomyosarcoma being the most common type in adults [11]. Small cell carcinomas of the urinary bladder are extremely rare, accounting for 0.35-0.70% of all bladder carcinomas [12]. Treatment options for different subtypes of NTCCB include surgery, radiation therapy, chemotherapy, immunotherapy, or a combination of these modalities. The choice of treatment depends on the tumor stage and the patient's overall health.

NTCCB present significant clinical challenges due to their rarity, heterogeneity, and poor prognosis. Compared to TCC of the bladder, NTCCB tend to be more aggressive and invasive and has a higher recurrence rate. They often present at an advanced stage, making treatment more challenging and adversely affecting patient outcomes. However, the exact prognosis depends on various factors, including the subtype of NTCCB, tumor stage, and grade, as well as the patient's overall health.

Despite the poor prognosis associated with NTCCB, the treatment has historically been based on the same principles used for TCC, which may not be effective for these rare malignancies. Therefore, there is a need for more targeted treatment approaches that consider the unique biology and behavior of NTCCB. Furthermore, the lack of standardized reporting systems and limited research on NTCCB have resulted in a lack of consensus on the optimal management of these malignancies. Hence, studying NTCCB is crucial for enhancing our understanding of the disease and developing more effective treatment strategies. This study presents our institutional experience over a decade in managing non-transitional cell carcinomas.

## Materials and methods

Study design and setting

The current study was a retrospective analysis of patients with NTCCB who presented at our hospital between January 2012 and December 2021. The inclusion criteria for the study were patients diagnosed with NTCCB based on histopathology reports. Patients with incomplete medical records, missing histopathology reports, or diagnosed with other concurrent primary malignancies with metastatic lesions in the bladder were excluded from the study.

Data collection and analysis

Patient data were collected from electronic medical records and paper charts of eligible patients. The collected data encompassed demographic information, clinical presentation, diagnostic workup, treatment details, and follow-up data. Descriptive statistics were utilized for data analysis. Categorical variables were expressed as frequency and percentages, while continuous variables were expressed as means ± standard deviation or median and range, as appropriate.

The demographic characteristics of the patients, including gender, presenting symptoms, history of other primary malignancies, other comorbidities, location of the tumor, stage at presentation, histopathological subtype, site of systemic metastasis, and primary treatment given, were analyzed descriptively. Frequencies and percentages were calculated for categorical variables, while continuous variables were summarized using means and standard deviations or medians and interquartile ranges, as appropriate. The tumor’s location was categorized into different subtypes, and the number of patients in each subtype was counted. Similarly, the histopathological subtype was also categorized, and the number of patients in each subtype was counted. The site of systemic metastasis was also recorded and analyzed. The median overall survival was determined by calculating the time from the initial diagnosis to the date of death, as recorded in hospital records or obtained through communication with the patient's relatives in cases where the death occurred outside the hospital setting.

Data were entered in Microsoft Excel spreadsheets, and statistical analysis was performed using SPSS version 22 (IBM Corp., Armonk, NY).

Ethical considerations and informed consent

This study was conducted by the ethical principles of the Declaration of Helsinki. The study protocol was reviewed and approved by the Institutional Ethics Committee. Informed consent was taken from the relatives of the patients after explaining the nature of the study and the benefits of the study results.

## Results

In our study, 31 patients were included, comprising 23 (74.2%) males and eight (25.8%) females. Hematuria was the most common presenting symptom reported by 23 (74.2%) patients, followed by suprapubic pain in seven (22.6%) patients and abdominal pain in one (3.2%) patient. Among the patients, 22 (71%) had no previous history of other primary malignancies. In comparison, nine (29%) had a history of diverse primary malignancies, including carcinoma colon, malignant vesicovaginal fistula, colonic adenomas and rectal polyps, carcinoma cervix, and carcinoma breast.

Comorbidities were observed in 13 (41.9%) patients. The most common location of the tumor within the bladder was the dome, accounting for seven (22.6%) cases, followed by the right lateral wall with six (19.4%) cases, and the posterior wall with or without trigone involvement with five (16.1%) patients. Other locations included the bladder base, diverticulum, left lateral wall, anterior wall, and multiple locations. Table 1 provides the descriptive statistics of patients diagnosed with NTCCB in our study.

**Table 1:** Descriptive statistics of patients diagnosed with NTCCB VVF: Vesicovaginal fistulae; NTCCB: Non-transitional cell carcinoma of the bladder

Variable	Number
Number of patients	31
Gender	
Male	23
Female	8
Presenting symptom	
Hematuria	23
Suprapubic pain	7
Abdominal pain	1
History of other primary malignancies	
None	22
Carcinoma colon	4
Malignant VVF	1
Colonic adenomas and rectal polyps	1
Carcinoma cervix	1
Carcinoma breast	1
Comorbidities	
Absent	18
Present	13
Location of tumor	
Dome	7
Right lateral wall	6
Posterior wall with or without trigone involvement	5
Bladder base	3
Bladder diverticulum	3
Left lateral wall	2
Anterior wall	1
Multiple locations	4

**Figure 1:**
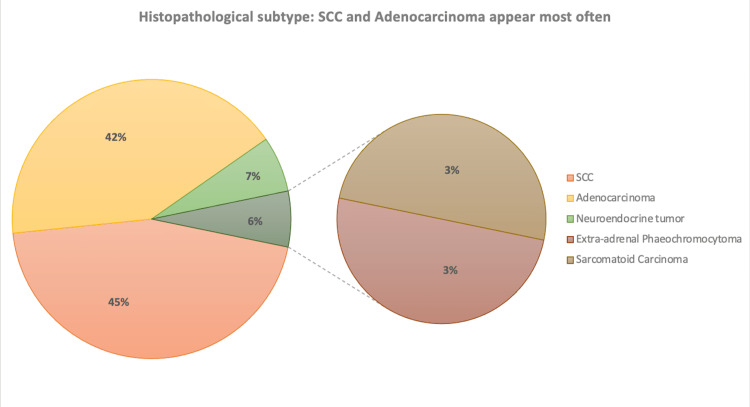
Diagrammatic representation of the different histopathological subtypes of NTCCB noted in our study SCC: Squamous cell carcinoma NTCCB: Non-transitional cell carcinoma of the bladder

Regarding the tumor stage at presentation, 15 (48.4%) patients had metastatic disease, 11 (35.5%) had localized tumors confined to the bladder, and five (16.1%) had locally advanced disease. The predominant histopathological subtype was SCC with 14 (45.2%) cases, followed by adenocarcinoma with 13 (41.9%) cases, neuroendocrine tumors with two (6.5%) cases, and extra-adrenal phaeochromocytoma and sarcomatoid carcinoma each with one (3.2%) case (Figure 1).

Among patients with metastatic disease, the most prevalent sites of systemic metastasis were the lungs affecting six (40%) patients, followed by liver and skeletal metastases, each found in three (20%) patients. Other sites of metastasis included the peritoneum, cerebral cortex, and non-regional lymph nodes.

**Figure 2:**
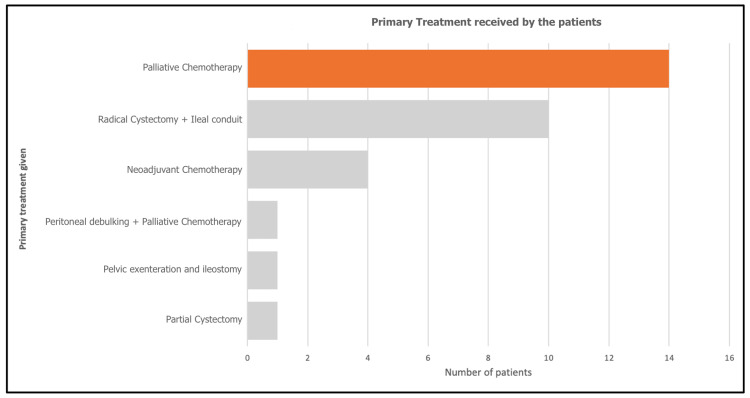
Primary treatment received by the patients diagnosed with NTCCB in our study NTCCB: Non-transitional cell carcinoma of the bladder

Primary treatment approaches varied, with 14 (45.2%) patients receiving palliative chemotherapy, 10 (32.3%) patients undergoing radical cystectomy combined with an ileal conduit, five (12.9%) patients receiving neoadjuvant chemotherapy alone, and one (3.2%) patient each undergoing partial cystectomy, pelvic exenteration with ileostomy, and peritoneal debulking with palliative chemotherapy (Figure 2).

Table 2 provides statistics on the tumor stage at diagnosis and primary treatment of patients diagnosed with NTCCB in our study.

**Table 2:** Descriptive statistics of tumor stage at diagnosis and treatment of patients diagnosed with NTCCB SCC: Squamous cell carcinoma; NTCCB: Non-transitional cell carcinoma of the bladder

The stage at presentation (Localized/metastatic)	Number
Metastatic	15
Localized	11
Locally advanced	5
Histopathological subtype	
SCC	14
Adenocarcinoma	13
Neuroendocrine tumor	2
Extra-adrenal phaeochromocytoma	1
Sarcomatoid carcinoma	1
Site of systemic metastasis	
Lung	6
Liver	3
Skeletal	3
Peritoneal	2
Cerebral cortex	1
Non-regional lymph nodes	1
Primary treatment given	
Palliative chemotherapy	14
Radical cystectomy + ileal conduit	10
Neoadjuvant chemotherapy only	4
Partial cystectomy	1
Pelvic exenteration and ileostomy	1
Peritoneal debulking + palliative chemotherapy	1

The median survival varied based on the disease stage and histopathological subtype. For localized SCC, the median survival was 32 months (57.1%), while for locally advanced SCC, it decreased to 13 months (92.9%). Patients with metastatic SCC had a median survival of 12 months (100%). Localized adenocarcinoma had a median survival of 39 months (15.4%), while locally advanced adenocarcinoma had a median survival of 15 months (7.7%). Among patients with metastatic adenocarcinoma, the median survival was 11 months (76.9%). Extra-adrenal phaeochromocytoma had a median survival of 57 months. Neuroendocrine tumors had a median survival of 10 months, and sarcomatoid carcinoma had a median survival of nine months. Table 3 presents the median survival of different subtypes of NTCCB, as noted in our study.

**Table 3:** Median survival of different subtypes of NTCCB in our study NTCCB: Non-transitional cell carcinoma of the bladder

NTCCB subtype	Median survival (months)
Squamous cell carcinoma (n=14)	
Localized (n=8)	32
Locally advanced (n=4)	13
Metastatic (n=2)	12
Adenocarcinoma (n=13)	
Localized (n=2)	39
Locally advanced (n=1)	15
Metastatic (n=10)	11
Extra-adrenal phaeochromocytoma (n=1)	57
Neuroendocrine tumor (n=2)	10
Sarcomatoid carcinoma (n=1)	9

## Discussion

NTCCB is a rare but aggressive form of bladder cancer with a poor prognosis. Our study noted a predominance of SCC and adenocarcinoma subtypes over other subtypes of non-transitional cell carcinoma bladder. Of the 31 patients diagnosed with non-transitional cell carcinoma bladder, 14 patients were found to have SCC, and 13 patients had adenocarcinoma. Only two patients had neuroendocrine tumors, one had sarcomatoid carcinoma, and one had extra-adrenal phaeochromocytoma. The combined prevalence of SCC and adenocarcinoma subtypes constituted our cohort's majority (87%) of NTCCB cases. Our study’s high incidence of SCC is consistent with previous reports suggesting that SCC is one of the most common subtypes of NTCCB worldwide, particularly in regions with a high prevalence of Schistosoma haematobium infection [13]. In our study, the etiology of SCC was not specifically evaluated. However, keeping in mind the non-endemic geography of India for Schistosoma haematobium, it can be assumed to be of non-bilharzial origin. On the other hand, adenocarcinoma of the bladder is a relatively uncommon subtype of bladder cancer, accounting for approximately 0.5-2% of all bladder tumors [14]. It has been reported to be associated with certain risk factors, such as bladder diverticula, urachal remnants, and long-term bladder catheterization [4]. Interestingly, the incidence of adenocarcinoma in our study was almost equivalent to that of SC. This high incidence of adenocarcinoma may reflect a selection bias, as our study was conducted in a tertiary care center with a high referral rate of complex cases. Most adenocarcinomas were metastatic at presentation, further adding to the possibility of delayed referral to our center.

Of the 31 patients with NTCCB included in our study, 15 had metastatic disease at the time of diagnosis, and five had locally advanced disease. The advanced stage of NTCCB at presentation, as noted in most of the patients in our study, has essential clinical implications as advanced-stage bladder cancer is associated with poor prognosis and limited treatment options. The advanced stage of NTCCB at presentation can be attributed to various factors, including the aggressive nature of the disease and the lack of specific symptoms in the early stages. Additionally, NTCCB subtypes have been shown to have more aggressive behavior and worse outcomes than transitional cell carcinoma. This may explain the high proportion of locally advanced and metastatic disease at the first presentation. The initial presentation of NTCCB at an advanced stage may limit treatment options and reduce the chances of curative treatment. In our study, patients with advanced-stage disease were more likely to receive palliative chemotherapy rather than curative treatment modalities, such as radical cystectomy. Furthermore, patients with metastatic disease had poor median survival rates, highlighting the need for improved treatment options for advanced-stage bladder cancer.

The median survival rates noted in our study for NTCCB subtypes vary widely. The median survival for locally advanced and metastatic SCC was 13 months and 12 months, respectively, with a median survival of 32 months noted in patients with localized SCC. Adenocarcinoma of the bladder had a median survival of 39 months in localized cases, 15 months in locally advanced cases, and 11 months in metastatic patients. Neuroendocrine tumors and sarcomatoid carcinoma had median survivals of 10 and nine months, respectively. Interestingly, the patient with extra-adrenal phaeochromocytoma of the bladder had the longest median survival of 57 months. However, it should be kept in mind that the patient had presented at an early stage which could have confounded the better survival associated with this diagnosis. Notably, these poor survival rates associated with NTCCB have significant implications for clinical practice. Firstly, it highlights the importance of early detection and prompt treatment for bladder cancer, as early-stage tumors have better survival rates than advanced tumors. Secondly, it emphasizes the need for tailored treatment approaches based on the histological subtype of non-transitional cell carcinoma bladder. For example, adenocarcinoma and SCC have different treatment algorithms and prognoses than transitional cell carcinoma. Therefore, identifying the histological subtype of bladder cancer is crucial for selecting appropriate management strategies and counseling patients on prognosis.

Overall, this study provides a valuable contribution to the limited literature on non-transitional cell carcinoma of the bladder and can serve as a starting point for future research to improve our understanding and management of this rare malignancy. The study can be helpful for future research in several ways. First, it provides a baseline for future studies to compare their findings. Researchers can use the information gathered in this study to design more targeted and specific studies focusing on particular subtypes of NTCCB or specific treatment modalities. Second, the study highlights the need for more extensive multi-center studies to understand better this rare malignancy's epidemiology, clinical presentation, and treatment outcomes. A larger sample size would allow for more robust statistical analyses and may reveal additional prognostic factors or treatment options. Third, the study identifies several areas where further research is needed, such as the role of immunotherapy and targeted therapy in NTCCB and the optimal management of metastatic disease. Future studies could address these questions by focusing on specific subtypes of NTCCB or conducting randomized controlled trials of novel treatments.

The strengths of our study include the rare nature of the non-transitional cell carcinoma subtype, which makes any data on this topic valuable. Additionally, our study provides insight into the clinical characteristics, management, and outcomes of patients with these rare subtypes of bladder cancer. The use of a retrospective study design allowed us to collect a significant amount of clinical data over a period of several years.

Our study has certain limitations that need to be taken into account. Firstly, the small sample size may restrict the generalizability of our findings to larger populations. Additionally, our study's single-center setting may limit the results' external validity to other clinical settings. Moreover, the retrospective design of our study may have introduced bias and limited the accuracy of the data collected. The absence of a standardized treatment protocol and different treatment modalities could have also influenced the observed outcomes. These limitations should be considered when interpreting the results of our study and in the design of future research studies aimed at evaluating the optimal treatment strategies for non-transitional cell carcinoma of the bladder.

## Conclusions

This study provides valuable insights into the clinical presentation, management, and outcomes of NTCCB. The findings underscore the importance of raising awareness and enhancing surveillance of this rare malignancy, which constitutes a small proportion of bladder cancers. NTCCB exhibits aggressive clinical behavior and presents nonspecific clinical features in the early stages, often leading to late diagnosis and an advanced tumor stage at presentation. Future multi-institutional studies with larger patient cohorts are needed to explore the optimal treatment strategies for NTCCB, to facilitate early detection and improve overall and cancer-specific survival rates.

## References

[1] Galsky MD, Iasonos A, Mironov S (2007). Prospective trial of ifosfamide, paclitaxel, and cisplatin in patients with advanced non-transitional cell carcinoma of the urothelial tract. Urology.

[2] Fortuny J, Kogevinas M, Chang-Claude J (1999). Tobacco, occupation and non-transitional-cell carcinoma of the bladder: an international case-control study. Int J Cancer.

[3] Bray F, Ren JS, Masuyer E, Ferlay J (2013). Global estimates of cancer prevalence for 27 sites in the adult population in 2008. Int J Cancer.

[4] Dahm P, Gschwend JE (2003). Malignant non-urothelial neoplasms of the urinary bladder: a review. Eur Urol.

[5] El-Bolkainy MN, Mokhtar NM, Ghoneim MA, Hussein MH (1981). The impact of schistosomiasis on the pathology of bladder carcinoma. Cancer.

[6] El-Sebaie M, Zaghloul MS, Howard G, Mokhtar A (2005). Squamous cell carcinoma of the bilharzial and non-bilharzial urinary bladder: a review of etiological features, natural history, and management. Int J Clin Oncol.

[7] Rausch S, Lotan Y, Youssef RF (2014). Squamous cell carcinogenesis and squamous cell carcinoma of the urinary bladder: a contemporary review with focus on nonbilharzial squamous cell carcinoma. Urol Oncol.

[8] Grignon DJ, Ro JY, Ayala AG, Johnson DE, Ordóñez NG (1991). Primary adenocarcinoma of the urinary bladder. A clinicopathologic analysis of 72 cases. Cancer.

[9] Roy S, Smith MA, Cieply KM, Acquafondata MB, Parwani AV (2012). Primary bladder adenocarcinoma versus metastatic colorectal adenocarcinoma: a persisting diagnostic challenge. Diagn Pathol.

[10] el-Mekresh MM, el-Baz MA, Abol-Enein H, Ghoneim MA (1998). Primary adenocarcinoma of the urinary bladder: a report of 185 cases. Br J Urol.

[11] Labanaris AP, Zugor V, Meyer B, Nützel R, Helmus S, Labanaris PG, Kühn R (2008). Urinary bladder leiomyosarcoma in adults. Int Urol Nephrol.

[12] Choong NW, Quevedo JF, Kaur JS (2005). Small cell carcinoma of the urinary bladder. The Mayo Clinic experience. Cancer.

[13] Arslan B, Bozkurt IH, Yonguc T (2015). Clinical features and outcomes of nontransitional cell carcinomas of the urinary bladder: Analysis of 125 cases. Urol Ann.

[14] Wilson TG, Pritchett TR, Lieskovsky G, Warner NE, Skinner DG (1991). Primary adenocarcinoma of bladder. Urology.

